# Blockade of the brain histamine H3 receptor by JNJ-39220675: preclinical PET studies with [^11^C]GSK189254 in anesthetized baboon

**DOI:** 10.1007/s00213-012-2733-x

**Published:** 2012-05-22

**Authors:** Jean Logan, Nicholas I. Carruthers, Michael A. Letavic, Steven Sands, Xiaohui Jiang, Colleen Shea, Lisa Muench, Youwen Xu, Pauline Carter, Payton King, Joanna S. Fowler

**Affiliations:** 1 Medical Department, Brookhaven National Laboratory, Upton, NY 11973 USA; 2 Johnson & Johnson Pharmaceutical Research & Development L.L.C., San Diego, CA 92121 USA; 3 National Institute of Alcohol Abuse and Alcoholism, Bethesda, MD 20852 USA

**Keywords:** Histamine H_3_, PET, JNJ-39220675

## Abstract

**Rationale:**

The preclinical characterization of a series of aryloxypyridine amides has identified JNJ-39220675 ((4-cyclobutyl-1,4-diazepan-1-yl)(6-(4-fluorophenoxy)pyridin-3-yl)methanone) as a high-affinity histamine H_3_ receptor antagonist and a candidate for further drug development particularly in the treatment of alcohol-related behaviors.

**Objective:**

This study measured brain histamine H_3_ receptor blockade by JNJ-39220675 (1 mg/kg) in the female baboon.

**Methods:**

Positron emission tomography imaging and [^11^C]GSK189254, a reversible high-affinity radiotracer with specificity for the histamine H_3_ receptor, was used to measure histamine H_3_ receptor availability at baseline and after i.v. and oral administration of JNJ-39220675 (1 mg/kg) in the anesthetized baboon. Histamine H_3_ receptor availability was estimated as the total distribution volume (*V*_T_) in brain regions. The sensitivity of [^11^C]GSK189254 binding to injected mass and carryover effects was determined.

**Results:**

JNJ-39220675 produces robust (ca. 90 %) blockade of [^11^C]GSK189254 binding after i.v. and oral administration. After oral administration of JNJ-39220675 (1 mg/kg), the fractional receptor occupancy was >0.9 at 90 min with a slight increase from 90 to 240 min. Similar to prior studies in humans, *V*_T_ was highly sensitive to the mass of GSK189254 with ED_50_ estimated to be 0.16 μg/kg.

**Conclusions:**

The robust blockade of binding of [^11^C]GSK189254 by JNJ-39220675 demonstrates that this compound readily penetrates the blood–brain barrier and occupies the histamine H_3_ receptor after oral administration at low plasma concentrations (∼1 ng/cc) supporting further drug development for alcohol addiction and other disorders. This study corroborates prior reports of the high sensitivity of [^11^C]GSK189254 to injected mass at doses >0.1 μg/kg.

## Introduction

Histamine is synthesized in histaminergic neurons which are localized in the tuberomamillary nucleus in the posterior hypothalamus and project widely throughout the central nervous system (Brown et al. 2001). The histamine H_3_ receptor is one of four histamine receptor subtypes (Hill et al. 1997). It is predominantly expressed in the brain, with the highest density in the basal ganglia as well as hippocampus and cortical areas (Martinez-Mir et al. 1990). Histamine H_3_ receptors have been identified as presynaptic autoreceptors on histamine neurons where they negatively regulate histamine release, thereby modulating histaminergic tone (Hill et al. 1997; Arrang et al. 1983). The H_3_ receptor is also a presynaptic heteroreceptor in nonhistamine-containing neurons in both the central and peripheral nervous systems. Nonhistamine-containing neurons modulated by the H_3_ receptor utilize a variety of neurotransmitters including gamma-aminobutyric acid, noradrenaline, acetylcholine, dopamine, and serotonin.

Histamine H_3_ receptors are involved in a variety of physiological functions including sleep/waking (arousal), motor activity, eating behavior, and cognitive function (Passani et al. 2004, 2011; Esbenshade et al. 2008). There is also evidence that antagonism of histamine H_3_ receptors will selectively produce enhanced neurotransmission in a variety of these neuronal systems and that the increase in transmitter turnover will have beneficial consequences for cognition, arousal, and a variety of other CNS processes (Witkin and Nelson 2004) as well as obesity and diabetes mellitus (Yoshimoto et al. 2006). Of particular interest is the potential for H_3_ antagonists in the treatment of alcoholism which is a major health problem worldwide with overall economic costs approaching $200 billion annually in USA alone. Three drugs in USA, disulfiram, naltrexaone, and acomposate, are approved for the treatment of alcoholism, but none of these are truly effective and all demonstrate high relapse rates. The cortico-mesolimbic area of the brain where alcohol and other drugs of abuse exert their reinforcing and rewarding effect is rich in histamine H_3_ receptors, and evidence suggests that the histaminergic system is involved in alcohol-induced behaviors via interactions with this system. Preclinical studies in rodents found high alcohol preference and low alcohol sensitivity correlated with brain histamine and H_3_-mediated processes (Panula and Nuutinen 2011). Thus the investigation of the neurobiology of the brain histamine H_3_ receptors as well as the therapeutic potential of drugs which target this relatively new molecular target has received considerable attention (Bonaventure et al. 2007) as has the development of methods for assessing their in vivo behavior in the setting of drug R&D.

**Fig. 1:**
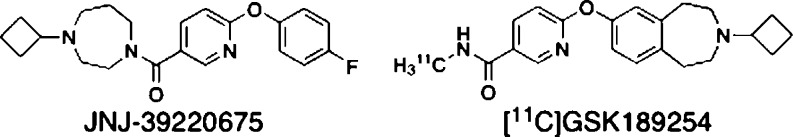
Structures of JNJ-39220675 and [^11^C]GSK189254

Letavic et al. (2010) recently reported the results of a preclinical characterization of a large series of aryloxypyridine amides and identified one of these ((4-cyclobutyl-1,4-diazepan-1-yl)(6-(4-fluorophenoxy)pyridin-3-yl)methanone, JNJ-39220675; Fig. 1a) as a high-affinity histamine H_3_ receptor antagonist (Ki, 1.4 nM). In addition to its clean receptor-binding profile, functional assays revealed that JNJ-39220675 significantly increased histamine levels in the frontal cortex consistent with histamine H_3_ receptor antagonism while also significantly increasing wake duration following s.c. administration in rats (Letavic et al. 2010). Support for the H_3_ alcohol link also comes from a recent report that JNJ-39220675 dose-dependently reduced both alcohol intake and preference in alcohol-preferring rats without changing alcohol pharmacokinetics (Galici et al. 2011). These studies support the further development of JNJ-39220675 as drug candidate and the current study to further characterize the behavior of a single dose of JNJ-39220675 with respect to in vivo BBB penetration and histamine H3 receptor occupancy in nonhuman primates.

[^11^C]GSK189254 (6-[(3-cyclobutyl-2,3,4,5-tetrahydro-1H-3-benzazepin-7-yl)oxy]-N-[^11^C]methyl-3-pyridinecarboxamide hydrochloride; Fig. 1b) is a high-affinity, reversibly binding radioligand which has been recently synthesized and validated as a radiotracer with specificity for the histamine H_3_ receptor both in pigs (Plisson et al. 2009) and in humans (Ashworth et al. 2010). Moreover, it is labeled with carbon-11 which has a 20.4-min half-life making it ideal for serial studies in which an individual animal or human subject can serve as a control for a subsequent drug treatment scan. However, the previous study of [^11^C]GSK189254 in humans has cautioned that its binding is highly sensitive to the levels of carrier GSK189254 present and that measures of histamine H_3_ receptor availability requires high-specific activity of the radiotracer in order to avoid receptor inhibition by the mass of the “tracer” itself (Ashworth et al. 2010). Here, we used the serial study design in the anesthetized female baboon to determine whether JNJ-39220675 administration inhibits [^11^C]GSK189254 binding in the brain. We also examined the effect of specific activity of [^11^C]GSK189254 on baseline measures of histamine H_3_ receptor availability in order to account for tracer mass and carryover effects.

## Materials and methods

### Study design

Six female baboons received 14 baseline scans with [^11^C]GSK189254. In one baboon (Reilly), the baseline was repeated on the same day. In a second (Lulu), the baseline scan was repeated on two different days 1 month apart; in a third (Kennedy), baseline scans were done 2.2 years apart. In three animals (Daisy, Friendly, and Kennedy), the baseline scan was followed by an intravenous dose of JNJ-39220675 administered 10 min prior to a second [^11^C]GSK189254 scan. In two animals (Spicey and Daisy), after a baseline scan, JNJ-39220675 (1 mg/kg) was administered via a nasogastric tube followed by the second and third scans at 90 and 240 min after the drug. For one of the studies in which JNJ-39220675 was administered orally, plasma samples were collected at baseline (to serve as a blank), at the time of the second PET scan, and at the end of the second scan 90 min later. The samples were centrifuged, frozen, and stored at −20 °C until analysis of drug levels.

### Drug administration

JNJ-39220675 (1 mg/kg) was dissolved in 5 % dextrose (1.2 mL 5 % dextrose/mg of JNJ-39220675) with warming and filtered through a 0.22-μ filter for intravenous administration. The solution was prepared in the same way for administration through the nasogastric tube but not filtered.

### [^11^C]GSK189254 preparation

[^11^C]GSK189254 was prepared by the alkylation of 6-[(3-cyclobutyl-2,3,4,5-tetrahydro-1H-3-benzazepin-7-yl)oxy-pyridine carboxamide by an adaptation of the literature procedure (Plisson et al. 2009). The precursor (0.5 mg) was dissolved in 0.2 mL of DMSO, and the mixture vortexed to dissolve it and 4 μL of tetrabutylammonium fluoride (1 M in THF with 4–7 % water). [^11^C]Methyl iodide, prepared from ^11^CO_2_ using a GEMS automated system (GE Healthcare) was passed through the solution in a stream of helium gas. The vessel was sealed and heated at 100 °C for 5 min. HPLC solvent (1 mL CH_3_CN/phosphate buffer (0.024 M, pH = 7.2 (27:73)) was added to the reaction vessel, and the solution was transferred onto semipreparative HPLC column (Phenomenex Gemini C18 (250 × 10 mm, 5 μm) and flow 4.5 mL/min). The precursor elutes at ∼14 min and the [^11^C]GSK189254 elutes at ∼19 min. The HPLC solvent is removed from the product fraction by rotary evaporation, and the product is dissolved in 5 mL saline and filtered through a 0.22-μm filter. Rapid TLC of the product cospotted with an authentic standard is run on Analtech UNIPLATE with methanol/ethyl acetate (1:1) (Rf = 55).

### Baboon preparation

Baboon studies were approved by the Brookhaven Institutional Animal Care and Use Committee. Adult female baboons (*Papio anubis*) (average weight 16.9 kg) were anesthetized with an intramuscular injection of ketamine hydrochloride (10 mg/kg), intubated, and for studies in which drug is administered orally, a nasogastric tube was installed. The baboon was transported to the PET laboratory and maintained on a gaseous mixture of oxygen, nitrous oxide, and isoflurane throughout the imaging session. Catheters were placed in an antecubital vein for radiotracer injection and for plasma sampling for drug quantification and in the radial artery for blood sampling for radiotracer quantification. EKG, blood pressure, O_2_ saturation, and respiratory settings were continuously monitored throughout the study.

### PET scans

For brain imaging, the head of the baboon was positioned at the center of the field of view as defined by imbedded laser lines. Scanning was performed on a high-resolution PET scanner (Siemens HR+, 63 slices, 4.5 × 4.5 × 4.8 mm) in 3D mode. A transmission scan is obtained with a ^68^Ge rotating source for attenuation correction of the emission data. For each PET study 1.01–4.8 mCi of [^11^C]GSK189254 was injected intravenously. The specific activity of the [^11^C]GSK189254 averaged 1.62 ± 1.83 Ci/μmol (range 0.15–5.04 Ci/μmol at time of radiotracer administration, 66 min post end of cyclotron bombardment) for the baseline scans. The mass injected averaged 2.84 ± 3.4 μg (range 0.073–13.0 μg). Emission data were collected in 3D mode for 90 min using a 35-frame acquisition sequence (1 × 10, 12 × 5, 1 × 20, 1 × 30, 8 × 60, 4 × 300, and 8 × 450 s frames). Arterial plasma samples were obtained throughout the time course of the study (every 2.5 s for the first 2 min (count every other one) and then at 5, 10, 20, 30, 60, and 90 min). Samples at 5, 10, 30, 60, and 90 min were analyzed for unchanged [^11^C]GSK189254.

### Plasma C-11 analysis

Arterial whole blood samples from either an automated blood sampling device (first 2 min) or hand-drawn samples were centrifuged for 1.5 min at 13,200 rpm in heparinized Eppendorf tubes. Plasma was aliquoted (typically 0.1–0.4 mL) by pipette from the spun samples, and radioactivity is assayed using a calibrated NaI well-type gamma counter. Plasma from six arterial whole blood samples (1, 5, 10, 30, 60, and 90 min after injection) is separated (as above), aliquoted (0.1–0.6 mL), and diluted with 3 mL of water. The diluted samples were then processed automatically by a commercially available laboratory robot (Zymark, Caliper Life Sciences) in the following manner. The diluted plasma was poured onto a preconditioned (5 mL methanol followed by 5 mL pH 7 phosphate buffer) solid-phase extraction cartridge (C18, 500 mg, Bond Elut LRC, Varian) preloaded with 2 mL of water and pushed into a clean test tube. The cartridge was then rinsed sequentially with 5 mL and 2 × 5 mL 60 % methanol/water, collecting each rinse fraction in a separate clean test tube. The radioactivity of all the rinse fractions, the total radioactivity in the sample tube, the C18 cartridge, and the empty sample tube was then assayed in a NaI well-type gamma counter. Data were corrected as described above, and the percentage of parent compound remaining at each sampling time was computed from the ratio of the C18 cartridge radioactivity to the sum of the radioactivity from all the rinse fractions and the cartridge. The fraction of total C-11 in the chemical form of [^11^C]GSK189254 at selected time points is used to correct the time–activity curve of total C-11 to generate the arterial input function which is used for the estimation of the total distribution volume *V*_T_ of each brain region.

### Image and data analysis

Time frames for the PET scans were summed from 0 to 90 min for the purpose of region of interest (ROI) placement. Regions of interest were placed over the striatum, cerebellum, frontal cortex, thalamus, cingulate, and temporal cortex, and a global region obtained by placing an ellipse over five central planes. ROIs are then projected back onto the dynamic frames to form the time–activity curves for each region.

Regional total distribution volumes (*V*_T_) were estimated using graphical analysis (Logan et al. 1990). In order to determine the possible effect of tracer mass on *V*_T_ estimates, the global total distribution volume (*V*_T_) vs the micrograms per kilogram (mg/kg) of injected tracer from 13 baseline studies was fit to the function as follows:

1 $$ {V_{\text{T}}} = {V_{\text{ND}}} + {V_{\text{S}}}/\left( {{1} + x/{\text{E}}{{\text{D}}_{{{5}0}}}} \right) $$

using SigmaPlot (*x* is in mg/kg; the fitting parameters are *V*_S_, the specific binding component; *V*_ND_, the non-displaceable (nonspecific) component of the distribution volume; and ED_50_, the dose that occupies 50 % of the receptors). *V*s is proportional to the number of free receptors (*B*_max_ if there is no binding due to endogenous ligand). In the absence of a mass effect, *V*_T_ = *V*_ND_ + *V*_S_. The corresponding occupancy (Occ) due to unlabelled tracer mass is given as:

2 $$ {\text{Occ}} = x/\left( {{\text{E}}{{\text{D}}_{{{5}0}}} + x} \right) $$

Using the values for *V*_ND_ and ED_50_, the true *V*_S_ can be determined from the measured mg/kg. This was done for all regions from the baseline studies. In the case of test/retest, the baseline study was corrected (using Eq. (1)), and the effective occupancy (Occ) of the second (retest) study was estimated using Eq. (3), known as a Lassen plot (Cunningham et al. 2010):

3 $$ V_{\text{T}}^{\text{Base}} - V_{\text{T}}^{{{\text Re} {\text{test}}}} = {\text{Occ}}\left( {V_{\text{T}}^{\text{Base}} - {V_{\text{ND}}}} \right) $$

where Occ is the sum of the occupancy due to mass of the second injection as well as the carryover mass from the first study. Using this occupancy, *V*_T_s from the retest study can be corrected and compared to the first study. For pretreatment studies with JNJ39220675, the occupancy for each blocking study was also estimated using a Lassen plot with the *V*_T_ baseline $V_{\text{T}}^{\text{Base}}$ corrected for injected tracer mass (where necessary with Eq. (1)):

4 $$ V_{\text{T}}^{\text{Base}} - V_{\text{T}}^{\text{Drug}} = {\text{Occ }}\left( {V_{\text{T}}^{\text{Base}} - {V_{\text{ND}}}} \right) $$

However this occupancy has three components: occupancy due to drug, to mass from the second injection, and to carryover mass from the first injection. Except for the case in which the tracer mass in both injections is very low, the actual drug occupancy will be an approximation.

The percentage change in *V*_T_ was calculated as $100\left( {V_{\text{T}}^{\text{Base}} - V_{\text{T}}^{\text{Drug}}} \right)/V_{\text{T}}^{\text{Base}}$ where $V_{\text{T}}^{\text{Base}}$ was corrected for tracer mass.

### JNJ-39220675 analysis

Plasma concentrations of JNJ-39220675 were determined using liquid chromatography/tandem mass spectrometry (LC-MS/MS) analysis. A 1 mg/mL stock solution of JNJ-39220675 was prepared in dimethyl sulfoxide and further diluted in acetonitrile (1:20, *v*/*v*) to yield a 50-μg/mL working solution. Five microliters of the working solution was spiked into 995 μL of blank baboon plasma to yield 250 ng/mL concentration of JNJ-39220675 in plasma, which was then serially diluted to 125, 25, 5, 1, and 0.2 ng/mL with blank baboon plasma. A 2-mL 96-well plate was used for sample preparation. A 50-μL aliquot of each standard and incurred plasma sample was mixed with 150 μL of acetonitrile containing JNJ-40579513 (50 ng/mL) as internal standard and was vortexed for 1 min to precipitate the plasma proteins. The quality control (QC) samples (2, 40, and 200 ng/mL) were prepared from the 50 μg/mL working solution in the same manner as above. The plate with the standards, QC samples, unknown samples, and plasma blanks was then centrifuged for 5 min at 6,100× *g*. The supernatant was diluted 1:1 with water before injection onto the LC-MS/MS system.

The LC-MS/MS system comprised of an API 4000 tandem mass spectrometry (Applied Biosystems, Foster City, CA), two LC-20AD pumps with a CBM-20AD controller (Shimadzu, Columbia, MD), a LEAP CTC HTS PAL autosampler (CTC Analytics, Carrboro, NC), and a switching valve (Valco, Huston, TX The chromatography) was performed on a Phenomenex Synergi, hydro-RP column (2 × 50 mm, 4 μm particle size) using a gradient elution method (0–95 % B over 1.0 min) at a flow rate of 0.7 mL/min. The mobile phase consisted of 10 mM ammonium acetate in water (A) and 0.1 % formic acid in acetonitrile/methanol (1:1, *v*/*v*) mixture (B). The samples were analyzed in the Multiple Reaction Monitoring scan mode with electrospray ionization. JNJ 39220675 was monitored for the following transition: 370.1 → 216.0. The data were processed using Analyst software (Applied Biosystems, Foster City, CA). The lower limit of quantification was 0.2 ng/mL.

## Results

### Baseline scans

The highest uptake of C-11 after [^11^C]GSK189254 administration occurred in the striatum with lower levels in frontal and temporal cortex consistent with results of Martinez-Mir et al. (1990). The lowest uptake occurred in the cerebellum. Low specific activity [^11^C]GSK189254 resulted in lower overall brain uptake with peak uptake occurring earlier and brain regions clearing over time. In contrast, with higher specific activity (and lower mass), C-11 steadily accumulates over the 90-min scanning period.

**Fig. 2:**
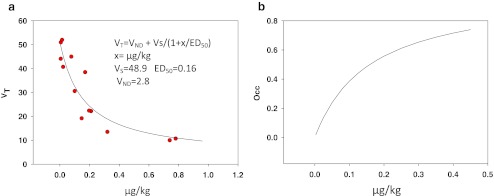
Effect of radiotracer mass of GSK189254 on distribution volume (*V*_T_) (**a**) global distribution volume vs μg/kg of tracer mass. The *solid line* is the fit to the model shown and (**b**) predicted histamine H_3_ occupancy based on the model of Eq. (2)

A plot of the global *V*_T_ vs injected dose in mg/kg for 13 baseline studies is shown in Fig. 2a. The solid line is the fit using Eq. (1) where ED_50_ = 0.16 μg/kg, *V*_ND_ = 2.8, and *V*_S_ = 49 mL/cm^3^. The predicted occupancy is shown in Fig. 2b. From this plot, [^11^C]GSK189254 binding is highly sensitive to injected mass even at doses less than 0.1 μg/kg. According to this data, a fractional occupancy of <0.1 would require the administration of <0.02 μg/kg.

**Fig. 3:**
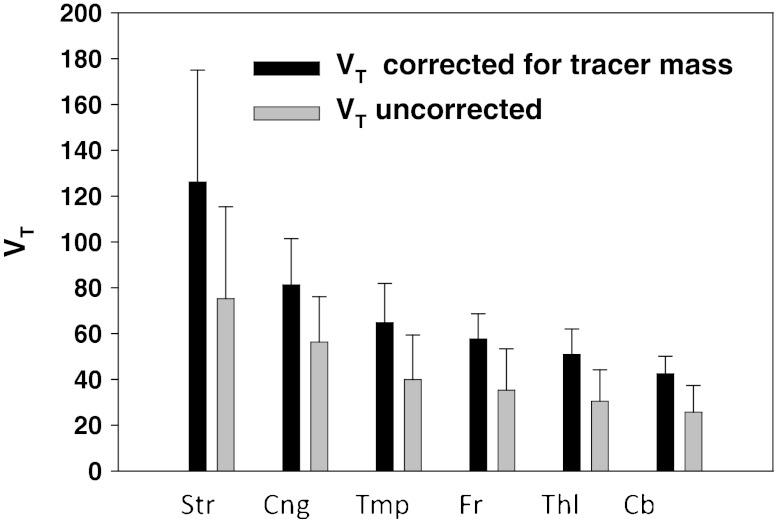
Average baseline *V*_T_s (*n* = 13) for striatum (*Str*), cingulate gyrus (*Cng*), temporal (*Tmp*), frontal (*Fr*), thalamus (*Thl*), and cerebellum (*Cb*) uncorrected and corrected for mass effects

Average baseline *V*_T_s for striatum (Str), cingulate gyrus (Cng), temporal (Tmp), frontal (Fr), thalamus (Thl), and cerebellum (Cb) are shown in Fig. 3, both corrected and uncorrected for mass effects. The ratio of *V*_S_ for Str to Cb is ∼3.1 (using the corrected *V*_T_s and assuming *V*_ND_ is on the order of three) so that the *B*_max_ in Str is approximately three times that in Cb.

A test/retest study on the same day in which the baseline and retest scans had injected masses of 0.78 and 0.33 μg/kg, respectively, had the same global *V*_T_, 10.7 vs 10.4 mL/cm^3^. This indicates a significant carryover mass in the second study since the predicted Occ’s for baseline and retest were 0.83 and 0.67, respectively, based on Eq. (1) which should have resulted in different *V*_T_s. This was verified using a Lassen plot (Cunningham et al. 2010). Correcting the baseline *V*_T_s for a mass of 0.78 μg/kg and using the retest *V*_T_s in Eq. (3) gives Occ = 0.87 for the retest study with 0.2 for the carryover occupancy. The average percentage change over six ROIs was −8.9 ± 4.9 % after correction compared to 7 ± 4.3 % before correction. The global and striatal *V*_T_s were 49.7 (test) and 50.0 mL/cm^3^ (retest), and 109 (test) and 113 mL/cm^3^(retest), respectively. The Lassen plot also provides an estimate of *V*_ND_, and this was found to be 4.4 mL/cm^3^ which is slightly higher than 2.8 mL/cm^3^ for the baboon group as a whole using Eq. (1). Repeating the calculation using *V*_ND_ = 4.4 mL/cm^3^ to correct the baseline scan gave the same occupancy for the second study. In another test/retest study (Lulu), 1 month apart with injected masses of 0.0065 and 0.022 μg/kg, the global and striatal *V*_T_s (corrected for mass as described above) were 47.5 (test) and 51.4 (retest) for global, and 118.5 (test) and 124 (retest) for striatal. The average percentage change over six regions was 2.8 ± 8.8 %. The test/retest, 2 years apart (Kennedy), had global *V*_T_s of 45 and 65 for the earlier and later scans (corrected for mass) and an average difference of −37 ± 0.5 %.

### Treatment with JNJ-39220675

**Fig. 4:**
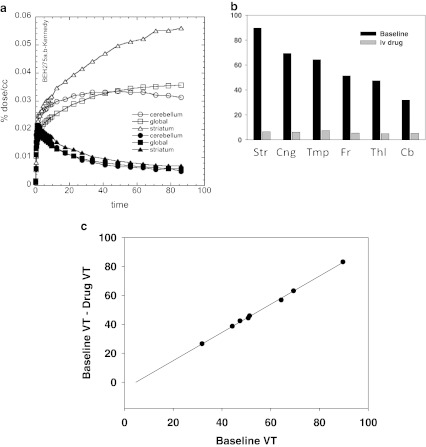
Time–activity curves and *V*_T_ for [^11^C]GSK189254 for one of the baboons for (**a**) the striatum, the cerebellum, and the global region at baseline and 10 min after i.v. administration of JNJ-39220675; injected mass of GSK189254 at baseline was 0.006 μg/kg. Note that after JNJ-39220675, time–activity curves for the different brain regions are indistinguishable. (**b**) Corresponding *V*_T_s at baseline and 10 min after JNJ-39220675 showing a significant reduction after drug (paired *t* test, *p* = 0.002). (**c**) Lassen plot for estimation of occupancy (0.97) and *V*_ND_ (4.6 mL/cm^3^). Data points are for the different regions of interest

The intravenous administration of JNJ-39220675 (1 mg/kg) in three different animals markedly reduced uptake of [^11^C]GSK189254 in all brain regions relative to a baseline scan consistent with robust blockade of baboon brain histamine H_3_ receptors. In these three studies, the injected masses of GSK189254 at baseline were 0.31, 0.74, and 0.006 μg/kg and 0.084, 0.18, and 0.007 μg/kg for the corresponding drug intervention scan. In the first two cases, the baseline scan must be corrected for mass, and the occupancy calculated for the drug study will contain a contribution from tracer mass. For the first two studies, the calculated fractional occupancies in the second scan were 0.97 and 0.94 with *V*_ND_s of 3.4 and 4.5 mL/cm^3^. However a substantial fraction of these occupancies is due to tracer mass in the second study. Uptake TACs and *V*_T_s for baseline and blocked high-specific activity study (0.006 μg/kg) are shown in Fig. 4a, b, respectively. The average decrease in *V*_T_ from baseline across brain regions was 87.8 ± 2.8 % (paired *t* = 7.2, *p* = 0.002). A Lassen plot for this study is shown in Fig. 4c. In this case, the fractional occupancy of 0.96 would be expected to be almost totally due to JNJ-39220675.

**Fig. 5:**
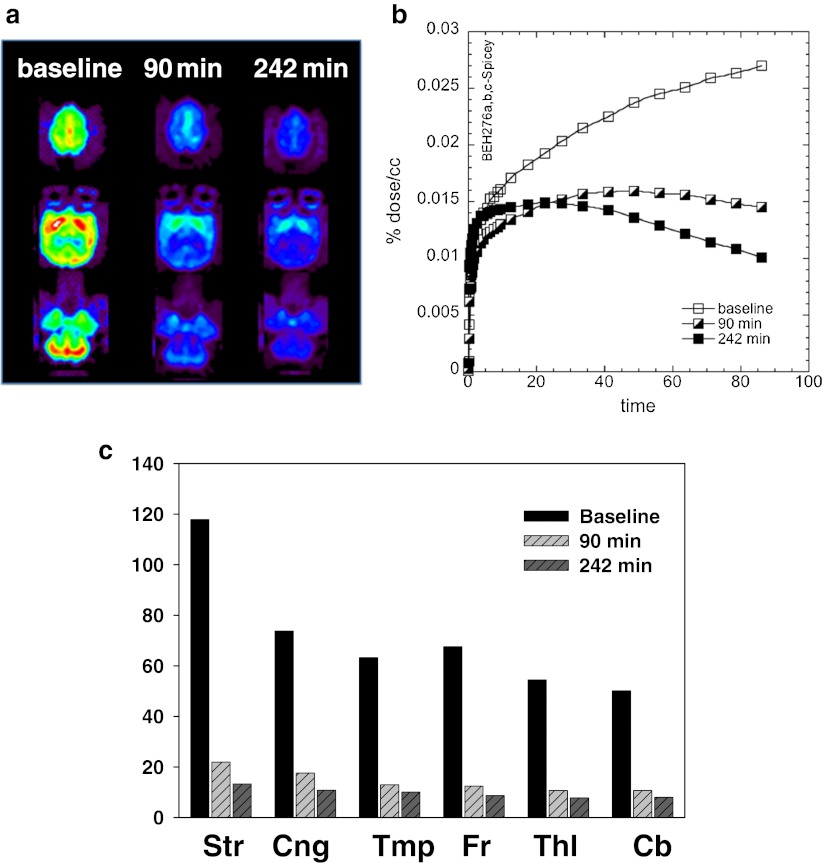
Effect of oral administration of JNJ 39220675 (1 mg/kg) on (**a**) PET images at the level of the striatum, (**b**) time–activity curves, and (**c**) corresponding *V*_T_s (baseline corrected for mass) for JNJ 39220675 administered via a nasogastric tube. A robust reduction of [^11^C]GSK189254 uptake is observed at 90 min and further reduction at 242 min (global *V*_T_s 57, 11.4, and 8 mL/cm^3^ for baseline for 90 and 242 min)

JNJ 39220675 administered via a nasogastric tube also produced a robust reduction of [^11^C]GSK189254 uptake and of the corresponding *V*_T_ in all brain regions in two animals scanned at 1.5 and 4 h post-administration. In these two studies, the injected masses of GSK189254 associated with the [^11^C]GSK189254 at baseline were 0.147 and 0.015 μg/kg. For the second study (0.015 μg/kg), the fractional occupancy was calculated to be 0.88 and 0.94 at 1.5 and 4 h for which the injected tracer masses were 0.005 and 0.003 μg/kg, respectively. The carryover mass should be low for these, so the occupancies reflect that of JNJ 39220675. Also the percentage reduction in *V*_T_ at 4 h was significantly greater than that at 1.5 h (*t* = 4.1, *p* < 0.01). Figure 5 shows PET images, TACs, and *V*_T_s for the three scans. For the other study the fractional occupancies were 0.98 at 1.5 and 4 h but the injected tracer masses were 0.78 and 0.43 μg/kg, respectively, so the occupancies most likely reflect both tracer and JNJ 39220675.

From the blocking studies and the test/retest study, the average non-displaceable component of the total distribution volume, *V*_ND_ from Lassen plots was found to be 4.78 ± 1.02 mL/cm^3^ which is somewhat higher than predicted by the model of Eq. (1). Using an exponential fit instead of Eq. (1) *V*_T_ = *V*_ND_ + *V*_S_ exp(−*x*/ED_50_) predicts *V*_ND_ = 8.1, *V*_S_ = 41 mL/cm^3^ (global), and ED_50_ = 0.14 μg/kg. The ED_50_s for both functions are close, but the exponential function appears to overestimate *V*_ND_, while Eq. (1) underestimates it. The average obtained from the baseline Lassen plots is in between at 4.7 mL/cm^3^. In all cases, use of a higher *V*_ND_ did not change the occupancy calculated using the Lassen plot. From these studies, we estimate that a 1-mg/kg dose of JNJ 39220675 administered either intravenously or orally blocks ∼90 % of [^11^C]GSK189254 specific binding in the anesthetized female baboon.

## Discussion

PET imaging with highly specific radiotracers is a safe, relatively noninvasive method to determine whether a drug enters the brain and engages a specific molecular target in humans and in animals in vivo (Nutt et al. 2007). PET has been applied to the development of new drugs (Fowler et al. 2010) as well as to the investigation of the pharmacokinetic and pharmacodynamic effects of approved drugs (Volkow et al. 2009). When the radiotracer is labeled with carbon-11, serial studies can be performed in the same individual (baseline PET scan and up to three more PET scans in a day), allowing an individual to serve as his/her own control and reducing the effect of intersubject and intrasubject variability (Volkow et al. 2005). When plasma drug levels are also measured, the relationship between drug PK and drug occupancy can be assessed, and when such a relationship is found, plasma drug levels can be used as a surrogate for drug occupancy in the brain (Fowler et al. 2010).

The main finding from this study is that the aryloxypyridine amide, JNJ-39220675, administered either intravenously or orally at a dose of 1 mg/kg enters the brain and produces robust blockade of brain histamine H_3_ receptors as determined by reduction of [^11^C]GSK189254 binding relative to a baseline scan and occupancy calculations. In this same study, we report the high sensitivity of [^11^C]GSK189254 binding to mass and carryover effects corroborating a recent report in humans (Ashworth et al. 2010). An in vitro comparison of GSK189254 (used as the tracer in this study) with JNJ-39220675 (used as the drug) indicates both to be potent functional antagonists at the human H_3_ receptor. For both compounds, Ki (pA2) values were found to be 0.12 nM (9.4) and 1.4 nM (10.4) for GSK189254 and JNJ-39220675, respectively (details of the assays are provided in Letavic et al. (2008) and Barbier et al. (2004)). Since JNJ-39220675 has not yet been labeled, details of the in vivo kinetics cannot be compared to GSK189254.

JNJ-39220675 inhibited >90 % of the binding of [^11^C]GSK189254 after i.v. or oral administration of a dose of 1 mg/kg. This is consistent with good blood–brain barrier penetration predicted by JNJ-39220675's physical properties (MW 369.44, log D 2.08) (Letavic et al. 2010). It is also consistent with its preclinical profile reporting that JNJ-39220675 has high affinity and high specificity for the histamine H_3_ receptor and that it occupies 90 % of histamine H_3_ receptors at a dose of 1 mg/kg in rats, mice, and dogs following oral administration (Letavic et al. 2010).

In the current study, a separate bioanalysis of the concentration of JNJ-39220675 at two time points after oral administration showed that the 90 % receptor occupancy corresponded to plasma concentrations of 1 and 1.3 ng/cc at the beginning and end of the second scan. We note that histamine H_3_ occupancy levels of 90 % effectively reduced alcohol intake and preference in a genetic model of human alcoholism, while an occupancy level of 75 % was not effective. This highlights the importance of accurate estimates of receptor occupancy vs dose in human clinical trials prior to interpreting clinical results.

For JNJ-39220675, we have previously demonstrated the relationship between receptor occupancy and histamine release in the frontal cortex of freely moving rats (Letavic et al. 2010). We observed that high receptor occupancy (approximately 90 %) was required for a statistically significant increase in histamine levels and that this correlated with EEG measurements showing a statistically significant increase in wakefulness. A similar relationship was observed for effects on cognition (unpublished). The correlation between receptor occupancy and wakefulness has also been reported for other histamine H_3_ receptor antagonists (Raddatz et al. 2012), and wake-promoting activity is only observed at doses producing greater than 80 % receptor occupancy (Letavic et al. 2010). Consequently, recognizing that the effect of a histamine H_3_ antagonist on alcohol intake and preference is due to changes in brain histamine concentration, and the relationship between histamine levels and histamine H_3_ receptor occupancy noted above, one would anticipate a requirement for high receptor occupancy for efficacy.

While the injected mass for a radiotracer for serial studies in the same individual in the same day are usually not subject to mass and carryover effects, the use of high-affinity radiotracers like [^11^C]GSK189254 (0.1 nM;(Plisson et al. 2009)) requires a special caution. Even at relatively low injected mass (<0.1 μg/kg of GSK189254), we saw a significant mass effect on baseline scans (Fig. 4) and dramatic reduction in *V*_T_ at doses >0.1 μg/kg. In fact, a prior study in humans reported that in order to occupy <5 % of histamine H_3_ receptors, no more than 0.003 μg/kg should be administered. Low injected mass (<1 μg/kg) could be achieved by long cyclotron irradiation and by limiting the amount of [^11^C]GSK189254 injected to 1 mCi in the baboon.

We note that even at the high dose of JNJ-39220675 administered in these studies, residual binding of [^11^C]GSK189254 remained. This non-displaceable binding (*V*_ND_) averaged 4.78 ± 1.02 mL of plasma/cm^3^ of tissue for the six brain regions and was accounted for in the estimation of receptor occupancy. For comparison, the *V*_ND_ of 10.54 ± 1.78 mL of plasma/cm^3^ of tissue has been reported for human dose–receptor occupancy studies with [^11^C]GSK189254 (Ashworth et al. 2010).

In summary, a growing number of structurally diverse chemical compounds with high specificity and high affinity for the histamine H_3_ receptor have been reported. The current PET study establishes mass requirements to minimize mass and carryover effects from [^11^C]GSK189254 for the anesthetized baboon, and this baseline data should be applicable to PET studies of other histamine H_3_ receptor active chemical compounds. It also extends the prior preclinical study of JNJ-39220675 (Letavic et al. 2010) and demonstrates good blood–brain barrier penetration and robust histamine H_3_ receptor blockade after i.v. and oral administration of a dose of 1 mg/kg.
